# Medical Association of Georgia

**Published:** 1898-05

**Authors:** 


### SOCIETY REPORTS.

### MEDICAL ASSOCIATION OF GEORGIA.

*Proceedings of the Forty-Ninth Annual Meeting, Held at Cumberland Island April 20, 21 and 22, 1898.*

The Association met at 2 p. m. in the auditorium of the Georgia Teachers' Association, and in the absence of the President, Dr. James B. Morgan of Augusta, the meeting was called to order by the First Vice-President, Dr. L. G. Hardman of Harmony Grove.

Col. R. E. Park was then introduced, and delivered an address of welcome on part of the Cumberland Island Club.

Dr. J. A. Butts, of Brunswick, welcomed the Association on part of the local profession.

The response to these addresses, in behalf of the Association, was made by Dr. Hardman.

Dr. J. G. Hopkins, of Thomasville, read a paper entitled "A Few Cases from My Note-book."

Case 1 was that of a man, thirty years of age, who complained of having "a chafe which would not heal." Upon examination the doctor found two fistulæ centrally located over the lower end of the sacrum, one inch apart, and about one-twelfth of an inch in diameter. He converted the two openings into one and found a bunch of curly blonde hair about two to two and a half inches long, and in amount sufficient to fill a No. 10 thimble. After scarification and cauterization, he left the wound to heal by granulation. A month later patient returned, saying that the wound had never healed. On examination the cavity was found well filled with new tissue, but there were several small fistulous openings instead of the two, as before. From each of these openings he drew several strands of hair similar to those first removed. He cut down again to the bone, drew a lot of hair out of the flesh from different directions and packed the wound. He again allowed the cavity to fill, and the same result was obtained. He again cut down and resorted to the same procedure, and the wound is now open and the same condition exists.

He said a dermoid cyst was not a very uncommon thing, but the sex, situation and constant recurrence of the hair in this case rendered it rather unique.

Another case was that of a mulatto, aged twenty-eight; personal history good; was never sick except with a slight attack of influenza in December, 1896. Was seen first on September 20, 1897. Had had fever about twenty-four hours; temperature 102° and pulse 128. Had a few bullæ on lips, face, wrists and shins. A diagnosis of pemphigus was made, and quinine and arsenic treatment begun. Local applications of dry boracic acid were resorted to. The bullæ appeared thick and fast, varying in dimensions from the size of a filbert to that of a walnut, and required but a few hours to reach full growth. They were punctured at each visit, giving exit to the fluid contents at the most dependent point, leaving the cuticle otherwise intact for protection. In a few days almost the entire cuticle had been separated from the cutis; the scalp, palms, and the mucous membrane of the nose, mouth and throat were extensively involved. The treatment while in the hospital was mercurial cathartics, followed by salines and liquor potassi arsenitis in increasing doses; also large doses of the sulphate of quinia. The local treatment consisted of alkaline baths, and a paste compound of aristol, bismuth, oxide of zinc and vaseline. Patient was enveloped in absorbent cotton from head to foot, the same being held in place by rolled bandages. His mouth and nose were kept clean with peroxide of hydrogen, and he was subjected to formaldehyde fumigation. The skin remained pigmented for three months after. Patient was discharged cured October 27, 1897.

Dr. William C. Lyle, of Augusta, followed with a paper entitled "The Importance of Careful Chemical Analysis in Gastric Disorders," in which he showed that the technique of chemical analysis of the gastric contents was by no means difficult, and that in the determination of a correct diagnosis it was of the utmost value. The skill to obtain and analyze the contents of the stomach did not lie beyond the dexterity and ability which every physician ought to possess, and it certainly should be utilized, when we consider that the diagnoses of diseases of the stomach are based as much upon the results of our own investigations as upon the statements of the patients themselves. The author contented himself by referring to only a few of the more important tests—tests requiring no apparatus nor reagents that the average practitioner does not possess. He first spoke of the test meal, then stomach contents, lactic acid, pepsin, and absorbent tests, after which he reported cases that had recently occurred in his practice, where a positive diagnosis would have been very difficult without frequent chemical tests.

The next paper, entitled "Germs of Health and Disease," was read by Dr. J. W. Duncan of Atlanta.

The author said that much attention had been given to the germ theory of disease for a number of years, and much had been learned through patient and faithful bacteriological research.

How can we most successfully limit the spread of contagious diseases? In the operating-room extensive major operations are being done daily under aseptic and antiseptic régime, and, notwithstanding the extensive field explored, not a microbe or bacteria can gain a foothold. Much is being done in private practice to limit the spread of the disease, but very much more should be done. The excreta from the sick, whether from the bowels, kidneys, lungs or skin, should be disinfected, and as soon as possible the house should be fumigated. It has been said that "cleanliness is next to Godliness," and therefore our sanitary boards should be vigilant in keeping everything clean—that is, in their province. There should be more attention given to the matter of the milk we drink, that there be no sources of contagion from them. Meats and vegetables demand investigation. Each individual should observe the laws of sanitation and hygiene relative to his own person and premises. By so doing he will be the better able to resist contagion should he be exposed.

Dr. W. H. Elliott, of Savannah, read a paper entitled "Mushrooms—A Food and a Poison." He stated that the number of mushroom-eaters in this country was steadily growing larger, and it was incumbent upon physicians to know how to recognize and treat cases of mushroom poisoning. Mushrooms belong to that order of plants known botanically as fungi. There are many thousand species. By far the larger number are microscopic, such as the mildew, the smut on grain, and the mold that forms on cooked fruit, etc. The larger forms of fungi are known indifferently as toadstools or mushrooms. These number about one thousand species. While mushrooms grow in many different forms, the commonest and best known is the campestris, or mushroom of commerce. This belongs to the class of agarics, or gilled mushrooms.

He spoke of three classes of mushrooms ordinarily used for food: agarics, boleti and puff-balls, and described their structures.

He said in the United States food was so abundant and mushrooms so little known that tons of delicious food were allowed to go to waste for the want of the gathering. Their use in this country was limited to a few botanists, and others, members of mushroom clubs of Boston, New York, Philadelphia and Washington. In Europe mushrooms were in general use as food. They were gathered in the woods and fields, regularly sold in the markets, and in Paris miles of cellars were maintained for the artificial cultivation of the mushroom of commerce. The essayist had found an interesting study in mushrooms. He had in the past two seasons tried about one hundred varieties of mushrooms without any unpleasant consequences. To the beginner he would say, avoid all mushrooms with a cup, or the suggestion of a cup, especially if the mushroom has white gills. Reject all that are not perfectly fresh, or that are worm-eaten. In short, mushrooms should be gathered with care and studied well.

Dr. Howard J. Williams, of Macon, read a paper entitled "A Supernumerary Cervical Rib; a Deception of Skiagraphy."

The author said that while the discovery of Roentgen was a material advance in surgical diagnosis, it was not an infallible aid. Mistakes in its interpretation could lead to mistakes in operating, and its value as medico-legal evidence was doubtful. Three times during the past year the speaker had been misled by X-ray photography; twice into performing useless operations for lesions supposed to be dependent upon apparent bone injury, and in one case a supposed tumor proved to be an anomalous rib. Fortunately, the operation was necessary, and the results were as satisfactory as they would have been had a tumor existed. The speaker thought that either of the two photographs of the bone injury exhibited in a court-room would have been damaging evidence in a malpractice suit, had not the operation disclosed the fallacy of the skiagraph. Dr. Williams then detailed the case of supernumerary cervical rib.

The patient pursued the usual normal course after aseptic operations, had primary union, and was restored to health without pain or interference with the functions of the parts involved.

Dr. Samuel Lloyd, of New York, said the use of the X-ray in medico-legal work, without careful preparation for that work on part of the surgeon, would do great harm. To leave a skiagraph to a single individual who might be interested in the case and go into court would be a grave error, for the deformity might be accentuated very decidedly by placing the tube in an improper position, and consequently in every case that has to appear in court the X-ray picture should be taken before witnesses who can swear to the position of the photographic plate, the position of the patient on the plate, and the position of the tube, and at the same time, who can swear to the distance at which the tube was placed from the patient, so that allowances may be made for any distortion that may appear in the picture, and as far as possible to avoid distortion.

Dr. T. S. Hopkins, of Thomasville, read a paper entitled "Dislocation of the Sixth and Seventh Cervical Vertebræ, with General Paralysis."

On the 3d of March, 1897, Col. H. sustained an injury, the results of which made the case not only interesting, but remarkable. The history of the accident is as follows: Patient while riding on a crank-car, the car collided with a wood cart. The shafts of the wood cart were elevated and one of them struck him on the forehead, knocking him over backwards, his neck falling across a thin iron bar. He was lifted from this position unconscious. At the end of an hour consciousness was restored. He was taken to the house of a friend, placed in bed, and medical aid called. Diagnosis of shock was made. Next morning a consultation was held, and the condition of the patient pronounced serious. On lifting him it was discovered that his head would fall over on his chest. It was also found that with the exception of the respiratory muscles, every muscle below the cervical part was paralyzed. Patient was then transferred to a Savannah hospital, where he recived the best medical skill, and three or four days after admission the neck trouble disappeared suddenly. The neck became the only unparalyzed portion of the patient. The diagnosis made by Dr. Boyd, the surgeon in charge, was dislocation of the sixth and seventh cervical vertebræ, with crushing of the cord, and sponstaneous reduction by muscular contraction. After three months of hospital treatment the patient was sent home as a hopeless case, the only improvement being a slight ability to move the right leg.

During the months of June and July last Dr. Hopkins frequently visited and examined the patient. He was on a very restricted diet, and there was much muscular waste and atrophy. He feared patient would never walk again. As he knew of no curative plaster to apply to a crushed cord, he advised that the restricted diet be abandoned, that physic be thrown to the dogs, and he be allowed to indulge in every article of food he could relish. This advice was carried out. Very soon the patient's dyspepsia disappeared; his appetite and digestion improved. He had beef, steak, ham, eggs, oysters, shrimps, crabs, fish, and every variety of vegetables and fruits. Under this treatment he gained flesh and strength. When he left him about the first of October last he had entire control of the right leg and arm. The index and middle fingers of the right hand were contracted. He could lift the left arm and hold it in any position except a perpendicular one, but the hand fell over on the wrist. This was not the result of contraction of the flexors, but the powerless condition of the extensor muscles. The bed sores had healed. The catheter and syringe which he had used for months were dispensed with, being no longer needed. The left leg was still paralyzed. Although the patellar reflex and motor power was absent in this leg, sensory power was present. Time and again he would say to the patient, "Lift this leg; you can if you will," but he always declared that it was impossible. Having observed in cases of locomotor ataxia that though the patient could hobble about with his eyes open, when he closed them he would fall, Dr. Hopkins determined to aid the muscular power with the visual power. He therefore uncovered the paralyzed limb so that the patient could see it, and commanded him to lift it, and instantly the leg was lifted, but fell again.

Some two weeks later patient's then condition was compared with his condition six months prior, and there was found to be decided improvement in the case. Patient was able to draw up both of his legs and extend them with perfect ease, has gained flesh and strength, and is looking much better. If he continues to improve in the next six months as he has during the past six months, he will be able to walk.

Dr. Samuel Lloyd, of New York, said that some ten years ago he published the statistics of over two hundred cases of operations upon the spine for injuries, and for Pott's disease, and since then he had operated on a considerable number of cases. In Dr. Hopkins's case he thought that one part of the diagnosis must have been in error, and that was with regard to crushing of the cord. A patient who has a crushed cord never recovers. The probabilities were that the cord was compressed, and from the progress of the case Dr. Lloyd suspected that the compression was a hemorrhage, and the absorption of the clot accounted for the recurrent power in the cord itself. Most assuredly there was no injury at the time of the receipt of the trauma that destroyed the elements of the cord itself, for had it been, the patient would have had an ascending and descending myelitis, and a consequent increase in the area of paralysis.

Dr. Hunter P. Cooper, of Atlanta, read a paper entitled "A Report of Surgical Cases." (To be published in this JOURNAL.)

Dr. Dunbar Roy, of Atlanta, contributed a paper on "Peritonsillar Abscess." (To be published in this JOURNAL.)

Dr. Willis F. Westmoreland, of Atlanta, reported twenty-nine successful cases of tracheotomy for foreign bodies in the air passages, and exhibited specimens.

Dr. M. F. Carson, of Griffin, followed with a paper entitled "The Condition of Imperfect Septum between the Mouth and Nasopharynx, Usually Termed Cleft Palate," in which he advocated early operation for the closure of the cleft.

Dr. J. H. Shorter, of Macon, read a paper on "Suppurative Diseases of the Middle Ear, and their Sequelæ."

The author said that otitis media purulenta may be acute or chronic, the latter often a sequence of the first; the former, as is the case in most affections, yields the more promptly to treatment, with altogether a better prognosis both as to cure of the suppurative process and restoration of the function in the diseased ear, while at the same time the risk of serious secondary complications was much less. The exciting cause of an otitis media acuta may be an influenza and acute pharyngitis, improper use of the nasal douche, sea-bathing, diphtheria, etc. It was particularly liable to occur in the course of exanthemata, especially scarlatina and sometimes is a complication of the other fevers, as pneumonia and typhoid. It may be traumatic, as from injury to the drumhead or injection of irritant liquids through the eustachian tube, a mode of treatment for chronic middle-ear catarrh favored by some men of eminence, but which he had quite abandoned on account of having seen so many cases of violent middle-ear reaction, and even suppuration set up by it, not alone in his personal practice, but in that of other surgeons, some of them of noted skill and experience. In New York there were always a large number of acute middle-ear inflammations during the season for surf-bathing. This he attributes, not as he first thought, to the impact of the sea waves on the side of the head, or the canal over the drum-membrane itself, but to the spasm accompanying the sneezing, swallowing and gagging caused by water entering the nostril, driving the liquid up the eustachian tubes into the tympanum. Among traumatic causes may be mentioned picking the ear with a sharp instrument for the removal of wax or a supposed foreign body. Not a few times he had known the drumhead to be lacerated by this foolish performance, and had a case on record where most of the membrane was removed, and with it the malleus and incus, done by a machinist who used a long awl in attempting to remove a supposed foreign body from the ear of a fellow workman. Predisposing causes are important factors in otitis media, both acute and chronic, and chief among them being obstruction in the nasal cavities or nasopharynx, adenoid or lymphoid tissue in the fold of the pharynx, hypertrophied faucial or pharyngeal tonsil, etc.

Dr. James M. Crawford, of Atlanta, read a paper on "Tonsillotomy; When and How to Make It."

Tonsillotomy should be resorted to when the tonsils extend much beyond the pillars, not waiting until they touch each other, especially when oral catarrh exists. It is indicated even when the tonsil is slightly hypertrophied if the lucunæ are inclined to inflammation from collections of caseous secretions. Shaving the tonsil destroys these lucanæ, thereby preventing frequent and painful inflammation. Not even the most timid operator would hesitate to make the operation when the tonsils are so large as to reach the uvula, thereby making it laborious to breathe, especially when the patient is asleep, and these diseased conditions often prevent respiration.

Below the age of fifteen the tonsil is usually soft, and when cut with the tonsillotome the cut edges are more or less mashed or pressed together, thereby stopping the hemorrhage. In older persons, however, the tonsil is more fibrous. The walls of the cut vessels are pulled apart, as it were, by the firmness of the tonsil itself. The speaker would not hesitate to make the operation when it was needed, even in the oldest. Fortunately, after a certain age the tonsils atrophy, and require to be removed rarely. Fewer cases of hemorrhage occur when the operation is made with the tonsillotome instead of the vulsellum forceps and bistoury. When using the forceps and bistoury the operator is apt to pull the tonsil with his forceps too much. In such a case he knows not where he is cutting. On completing the operation and looking into the mouth of the patient, he sees a sulcus where a portion of the tonsil should have been left. A sulcus, besides endangering the life of the patient by hemorrhage, is the source of constant annoyance in that it is a lodging place for food. The author prefers McKenzie's tonsillotome, or some of its modifications, to all others, for the reason that it is more simple, more easily managed and less cumbersome. There need be no fear of the tonsil falling into the larynx, as it nearly always adheres to the instrument. Where it does not adhere to the instrument it falls in the mouth and is expelled. By applying a little cocaine, say a 6 per cent. solution, to the tonsils and fauces, sensibility of the throat is allayed, which is a great aid in the operation.

While acting as assistant to Dr. Calhoun, he could recall five or six cases of frightful hemorrhage from tonsillotomy made by him with the knife and forceps. In each case the hemorrhage was stopped by Dr. Crawford putting a wet sponge or pledget of cotton on the cut surface and applying pressure. In each of these cases a sulcus existed, making it impossible for him to see the bleeding artery, and forcing him to resort to the only safe method in such cases, namely, pressure. The first time he used this method for controlling hemorrhage was in the fall of 1889. The two means for arresting hemorrhage in connection with tonsillotomy were the cautery, when the bleeding point could be seen, and in other cases the application of pressure.

Dr. W. Z. Holliday, of Augusta, read a paper on "The Use of Ethyl Chloride as a Local Anesthetic," in which he recommended this agent very highly for local anesthesia.

Dr. R. M. Harbin had used ethyl chloride, and agreed with the author as to its efficacy. In selected cases it was an ideal local anesthetic.

Dr. J. G. Hopkins asked as to the danger in handling the tubes, to which Dr. Holliday replied that there was very little danger from explosion if the physician was careful.

Dr. Graham, of Savannah, had used with satisfaction the combined method of anesthesia, namely, ethyl chloride and the infiltration method of Schleich.

Dr. J. T. Ross, of Macon, reported an interesting case of "Ossific and Calcified Ovarian Fibroma," and exhibited the specimen. The specimen had very much the appearance of a fetal head. On one side a portion of the broad ligament, the Fallopian tube, and ovary were seen. Almost, if not all, of the fibroid enlargement was encrusted by an osseous deposit about one-sixteenth of an inch in diameter. The interior of the tumor was more firm and studded with calcareous deposits. Striæ could be seen running down into the tumor from that portion of the ovary which formed the growth. Authorities are agreed that only three to five per cent. of all tumors of the ovary are solid, and that a fibroid of the ovary is very rare. The condition presented in the case of Dr. Ross was still more rare. In fact, the speaker does not remember to have seen a record of an ovary which had undergone both osseous and calcareous degeneration.

Dr. K. P. Moore, of Macon, read a paper entitled "A very Interesting and Unusual Monstrosity in the Form of Twins," and exhibited the feti.

Dr. E. R. Corson, of Savannah, contributed a paper, "A Rare Form of Bone Atrophy Following an Ununited Fracture, as Seen by the X-ray."

Dr. J. I. Griffith, of Danielsville, read a paper on "Puerperal Eclampsia, and Some Probable Causes of It." He had been stimulated to present a paper of the number of cases of puerperal eclampsia which had recently come under his observation.

In order to arrive at an intelligent treatment there must be a clear understanding of the condition the physician is called upon to treat. The treatment should be classified as preventive and curative. The preventive may be subdivided into medicinal and hygienic, and the curative into medicinal and obstetric. These forms of treatment were then dwelt upon at some length. The author reported four cases. Some probable causes of eclampsia he mentioned as albuminuria, toxemia, uremia, pyelitis and pyelo-nephritis. He thinks albumin is one of the causes of puerperal eclampsia, for it is found in the urine of pregnant women.

Dr. George H. Noble, of Atlanta, made some remarks on "Alcoholic Irrigation in Puerperal Sepsis." He pointed out the difference between the ordinary method of removing the secundines with douches and the alcoholic treatment. He said that the alcoholic treatment was gradually forcing itself upon the profession. When his attention was first called to it he had little or no confidence in it, for the reason that he did not believe in the antiseptic properties of alcohol, but the more he studied it the greater was its need of application in certain directions. The successful treatment of puerperal infection depended upon the selection of cases. The practitioner who failed to properly select his cases and properly apply treatment would be the man who would lose patients. No treatment applied to the cavity of the uterus could be expected to save a case of puerperal infection that had gone beyond the endometrium to any extent. Therefore it was necessary to exclude diseases of the appendages, as pus tubes, abscesses, etc. The treatment of the cavity of the uterus where there was a pus collection in the appendages was futile. Again, the practitioner might think the appendages were absolutely healthy, that there was no infection in the uterine cavity that could be detected, and yet the patient was in a hopeless condition, whereas the trouble may be an abscess in the uterus proper, or its parenchymatous structure. The practitioner should bear in mind that if he has a case in which appendages and peritoneum are not involved, and in which there is no material discharge from the cavity of the uterus, where the uterus is shrunken in size, the os non-patulous, he is likely to have uterine abscess, but it is a rare condition. There may be one abscess or two. He had previously reported four such cases in which he made an incision into the abscess, curetted, cauterized and drained through the abdominal cavity.

He said that alcoholic irrigation could be done by the general practitioner. The practitioner should use a rubber catheter, thoroughly sterilized; take two yards of small gauze, the width of the finger, stitch it to the end of the catheter, introduce it carefully into the uterus, after it is cleansed, and loosely insert the gauze around it. The object of the gauze is to hold the alcohol. The alcohol is renewed through the end of the catheter at variable intervals. Alcohol possesses considerable antiseptic properties, and has some inhibitory effect upon the spores of the virulent streptococci. Alcohol acts more powerfully where there is a good deal of water in the tissues. He believes it is the simplest method that he knows of for treating puerperal infection confined to the cavity of the uterus. It must be remembered, however, that all local treatment must be confined to those cases in which the infection is limited to the uterine cavity.

Dr. W. E. Fitch, of Savannah, contributed a paper on "Tight Lacing—Its Relation to Uterine Development and the Diseases of the Female Organs of Generation," in which he called attention to the evil effects of tight lacing. The corset was so constructed that when worn it exerts its greatest influence (pressure) from above the brim of the pelvis downward, constricting the abdominal walls, the lower part of the thorax, and pushing inward the costal cartilages, and often the seventh and eighth overlapping. The greatest constriction occurs in the immediate neighborhood of the stomach, which, when distended as after a hearty meal, produces the hourglass stomach found at times in this class of patients. Compression is so great in most cases as to interfere with the normal peristaltic action of the intestines, thereby producing constipation. Compression in any part interfered with physiological functions, and therefore the author arrived at the following conclusions:

1. The normal breathing of woman is like that of man—abdominal. Tight lacing changes the type to costal.

2. The pelvic organs normally make a considerable excursion with each respiration. Tight lacing in the upright position checks this motion almost entirely.

3. Sitting or leaning forward lessens intra-abdominal pressure. Tight lacing in these positions greatly increases intra-abdominal pressure.

4. The uterus is displaced downward by tight lacing from one to two and a half inches. The pelvic floor is bulged downward and the circulation rendered sluggish.

5. Uterine development is greatest from the twelfth to the sixteenth years. Tight lacing is usually commenced at this, the period of the beginning of uterine development.

Dr. J. L. Hiers, of Savannah, read a paper on "Malarial Asthenopia."

Dr. A. A. Davidson, of Augusta, read a paper on "Hysteria." Hysteria, he said, was a disease wherein the emotional is in the ascendancy over the volitional, and characterized by marked and marvelous expressions of the propensities of the idiosyncrasy of the person affected, and was not hysteria necessarily because of any abnormal condition of the uterus, but was a manifestation of a morbid and incoordinate activity of brain and nerve forces, volition being subordinated, characterized by symptoms motor, sensory and sympathetic, abnormal in nature. In expression it frequently simulated symptoms of various pathological and diseased states, such as hyperesthesia, anesthesia, paralysis, convulsions, general or local suspension of functions, secretory and excretory, etc.

The causes of this condition are predisposing and immediate or exciting. Heredity stands among the first of the former. Family history of phthisical, strumous or neurotic antecedents may confidently be sought; many conditions of unsatisfied nature, long mental worry and physical strain and protracted suspense.

In the treatment of hysteria the thing of first importance is to obtain the confidence of the patient that his or her symptoms have been carefully sought out and weighed, that the promise of complete recovery may be held out as the result of careful study of the case, else little can be effected. In seeking the mode of management of this disorder we look away from drugs except in case of complicating intercurrent diseases. This is the rule and principle, but there arise conditions at times which must be met by the energetic use of active medicinal agents. One cannot in every instance douse a pail of ice-water in the face of a patient if convulsions be present, though they be hysterical. The patient is a lady of social standing, of integrity and of delicate constitution, what is to be done? Power of moral suasion is not applicable by reason of the patient being in a semi-comatose, if not pseudo-comatose state, and will not be thus reached. Eminent writers would eschew such antispasmodics as bromides or chlorals. Experience leads him to the conclusion that where sleep can be induced and maintained for some hours a great advantage is gained. This can be effected by using chloroform to offset the paroxysm, continuing the sleep by the use of chloral per rectum. The subject wants to be taking medicine, and so reconstructives, tonics, etc., may very well be administered, since malnutrition, debility and anemia are favorable to the development and aggravation of hysterical symptoms.

A plan of treatment given by the author is that adopted by Dr. Weir Mitchell, of Philadelphia, introduced into this country by Dr. Playfair, which consists of complete isolation of the patient and maintenance in bed, milk being given in increasing quantities. Massage is used in lieu of exercise. Brilliant results have thus been secured. However, the plan is rarely practicable. If it be impossible to place the patient among non-apprehensive and unsympathetic attendants, for patient's sake and theirs, the family or those immediately interested should be acquainted with the true condition and educated to deal intelligently with hysterical attacks and phases. Parents should know what their impressionable children read; unlimited open air exercise should be encouraged. The mind should be engaged and kept off the imagined condition of self. The cardinal motive in the treatment should be to so environ the patient as to call forth no expressions of an emotional nature, but rather conduce to exercise of reason and will.

"Indications for and Antiseptic Technique of Uterine Drainage after Labor and Abortion." This was the title of a paper by Dr. R. R. Kime, of Atlanta.

In the treatment of puerperal infection, the practitioner must consider the anatomical relations and physiological functions of the female generative organs. The author does not believe that all cases of puerperal infection are due to contamination by physician or nurse, nor that the physician can always prevent such infection. We have sufficient clinical evidence to prove that where a uterus fails to properly drain itself, remaining large and flabby, a blood clot, portions of placenta, or a cotyledon is retained, infection occurs from putrefaction and absorption of uterine contents. Such cases are usually sapremia or putrid infection, but may be a mixed or true septic infection, due to the presence of septic germs in the genital tract previous to confinement. In cases of septic infection the author believes that the curette and gauze tampon have killed more patients than they have saved. When an active puerperal septic condition exists long enough to produce constitutional and local symptoms and signs sufficient to establish a diagnosis, the curette cannot reach the diseased parts on account of the germs having extended beyond the endometrium into the uterine walls, blood-vessels and lymphatics, and, in rapid septic cases, has extended so far that even hysterectomy is not justifiable in but few instances. He cares not if small portions of adherent placenta or cotyledon be present, efficient drainage will eliminate the toxins sufficient to wait for nature to separate these structures far more efficiently than the curette, when they can easily be removed by forceps without traumatism of the parts and with greater safety to the patient. The gauze tampon should never be used in a puerperal septic uterus except to check hemorrhage. So far as the present treatment is concerned, he considers uterine and alimentary drainage and elimination the most potent factors at our command. Uterine drainage is secured by use of drainage-tubes and strips of gauze or wicking. The tube should be removed and uterus irrigated once or twice in twenty-four hours in severe cases, being governed by pulse and temperature. If they rise it is an indication for irrigation or that the drainage is obstructed.

As to drainage in cases of incomplete abortion, there are different conditions to deal with. The author limits the term abortion to interruption of gestation at any time prior to the complete formation of placenta. At least ninety per cent, or more of cases of infection occurring during or after abortion are putrid infection, hence easier controlled and less demand for drainage. If an active septic infection occurs, then drainage is demanded in cases of abortion. The dangers and contraindications to the use of the curette and tampon in septic cases increase in proportion to the advance in pregnancy and the increase in size and vascularity of the pelvic organs. While the author advocates an antiseptic gauze uterine tampon after curetting the uterus in cases of abortion, it is to act as surgical dressing, prevent further infection, check hemorrhage, stimulate uterine contractions, and not for the purpose of drainage. The tampon should be removed in twenty-four to forty-eight hours, and not repeated. If after gauze is removed there is elevation of pulse and temperature with constitutional and pelvic disturbances, then drainage and elimination are indicated.

The following officers were elected for the ensuing year: *President*—Dr. Howard J. Williams, Macon; *First Vice-President*—Dr. J. G. Hopkins, Thomasville; *Second Vice-President*—Dr. I. H. Goss, Athens.

Place of meeting—Macon; time, third Tuesday in April, 1899.

---

Oletimer—Is your married life one grand sweet song?

Newlywed—Well, since I got a baby it's more like a grand opera, with loud calls for the author every night.—*Puck.*

